# The Metabolic Clock Model of B Cell Activation and Differentiation

**DOI:** 10.20900/immunometab20210019

**Published:** 2021-05-13

**Authors:** Munir Akkaya

**Affiliations:** Laboratory of Immunogenetics, NIAID, NIH Rockville, MD 20852, USA

**Keywords:** B cells, humoral immunity, peripheral tolerance

## Abstract

Recent advancements in the field of B cell immunometabolism have provided mechanistic insights to B cell activation and fate determination. Here, in this short article, I will explain the main principles of our novel metabolic clock model and how it may reshape our perspective on longstanding immunological questions related to pathologies arising from out of context B cell activation.

B cells are a cornerstone of adaptive immunity. They play critical roles not only in the elimination of acute infectious threats by rapid production of protective antibodies but also in preventing future encounters with the same pathogens through generation of memory B cells (MBCs) and long-lived plasma cells (LL-PCs) [1,2]. These highly differentiated descendants of naïve B cells are the main forces that provide long-term protection in response to nearly all vaccines that are currently protecting billions of people from deadly diseases. What is even more astonishing is that this protective machinery arises from the naïve B cell repertoire in our body which consists of 10^9^ newly maturated B cells entering circulation from bone marrow every single day [3]. Naïve B cells that do not encounter antigen challenge perish after a few weeks of patrol duty in the blood and lymphatic system. This rapid turnover and mass production of naïve B cells ensure diversity in the adaptive immune response. However, it also increases the likelihood of disorders that originate from “manufacturing issues” such as hematologic neoplasms and autoimmune disorders which stem from B cells that survive and proliferate out of context. Therefore, the immune system requires quality controls that ensure only non-self-reactive B cells are allowed to respond and only when challenged by a genuine antigenic treat.

Potentially hazardous self-reactive B cell clones that arise due to random receptor recombination events during development is predominantly eliminated in the bone marrow through various mechanisms in a process called central tolerance [4]. However, central tolerance is not at all perfect and significant numbers of weakly self-reactive B cells still complete their maturation and exit bone marrow into the periphery. These cells need to be destroyed or at least kept silent through the next level of regulatory mechanisms collectively known as peripheral immune tolerance [5,6]. A well-known strategy that is employed by peripheral tolerance in order to prevent out of context B cell activation is the requirement of two spatiotemporally distinct signals for induction of B cell activation. The first signal (signal one) comes from the encounter of B cell antigen receptor (BCR) with the antigen it is specific for. This wakes up the naïve B cell and alerts it for the likely possibility that a foreign pathogenic intruder is in the vicinity. However, signal one alone is often not enough to burn the bridges and embark on a full-scale immune activation. Therefore, a second signal (signal two), coming from either antigen-specific B cell-T cell interactions or through innate pattern recognition receptors (PRRs) such as Toll like receptors (TLRs), is required for the “go” order [3]. Provided that the T cell compartment is free of self-reactive T cells (which it appears to be) a successful B cell-T cell synapse confirms that the antigen the B cell has processed and presented to the T cell is not a self-antigen. Alternatively, microbial pathogen associated molecular patterns (PAMPs) hitting PRRs in B cells communicates to the B cell that the antigen the B cell encountered has a high probability of coming from the PAMP-producing pathogen. Only when B cell receives a “go” signal from either source, is full-scale immune activation kicked off.

Although the two signal hypothesis for B cell activation was embraced in the 1960s, the machinery behind it is relatively less characterized. Our recent studies of the metabolic and cellular alteration in naïve B cells triggered by signal one and signal two revealed a unique metabolic regulation machinery that controls the integration of the two signals in B cells [7–10]. We showed that the signaling cascades initiated by the binding of the antigen to the BCR lead to rapid increases in cellular respiration, nutrient uptake and expression of various activation markers. These early changes prepare the B cell to further differentiate into antibody-producing PCs. However, unless a second, independent stimulus is received soon after the antigen binding, BCR signaling does not lead to B cell proliferation and differentiation. Instead, in the absence of a second signal, antigen binding to the BCR leads to a dysregulation in cellular calcium homeostasis which in turn leads to mitochondrial dysfunction and a phenomenon termed activation-induced cell death (Figure 1). Our experiments in naïve murine B cells showed that there is approximately a 9 hour window during which B cells that are partially activated through signal one can receive signal two and be rescued from ionic dysregulation and mitochondrial dysfunction. If signal two is delayed beyond this limit, antigen triggered leak of calcium into cytosol continues and leads to calcium toxicity. This results in a mitochondrial pathology manifested with increased mitochondrial permeability, mitochondrial matrix swelling, loss of potential across inner mitochondrial membrane and thus inefficient oxidative phosphorylation leading to high levels of reactive oxygen species (ROS) production. Elevated ROS amplifies the mitochondrial pathology further by inducing more matrix swelling, ultimately leading to a total shutdown of mitochondria and B cell death. Therefore, we proposed that a “metabolic clock” initiated by signal one ticks towards metabolic death of the B cell unless it is deactivated by signal two in a timely fashion. While delayed signal two mediated rescue is possible, it is partial and less efficient. Our metabolic clock model provided a novel perspective on how B cell activation is controlled in the periphery. Although our current understanding is limited to cell survival vs cell death as two outcomes of the metabolic clock, with the new insights provided by the model, I hypothesize that cell survival and cell death may not be the only two outcomes of the interplay between signal one and signal two. An imbalance between signal one and two may actually create a spectrum of abnormal phenotypes depending on the strength and spatiotemporal qualities of the signals involved. Of particular interest is the possibility that the metabolic clock controls anergy of self-reactive B cells and atypical B cells [11] that arise in chronic diseases. If so, signal two may be more than a simple “go-no-go” signal for antigen-activated B cells. Rather signal two, depending on its nature, may serve as a checkpoint in peripheral tolerance and determine the fate of B cells differentiating in chronic diseases. Although B cell-T cell interactions are the gold standard to confirm that the antigen the B cell encountered is not a self-antigen, it is a time-consuming process requiring processing and presentation of the antigen that may not be well suited for acute systemic infections in which quick responses are critical for survival of the host. Although T cell- independent forms of signal two coming from innate PRRs might be handy, quick life-saving alternatives to T cell help when the time is of essence, the response to PRRs is only a bet on the likelihood that the antigen the B cell encountered and PAMP come from the same pathogen. Therefore, this quick solution may act as a double edged sword in which self-antigen bound anergic B cells that are kept silent due to the lack of signal two may receive an out of context kiss of life that revitalizes them by reversing their anergic states and may potentially direct them towards the path of harmful autoimmune conditions that are known to sometimes arise as consequences of infections [12–15]. Clearly, comprehensive experiments will be required to understand the nuts and bolts of the interplay between signal one and signal two before any link between infections and autoimmunity can be explained through principles of the metabolic clock model. Nonetheless, it is obvious that the advancements in the field of B cell immunometabolism will further our understanding of B cell biology and potentially offer novel explanations to longstanding questions in the field.

## Figures and Tables

**Figure 1.**
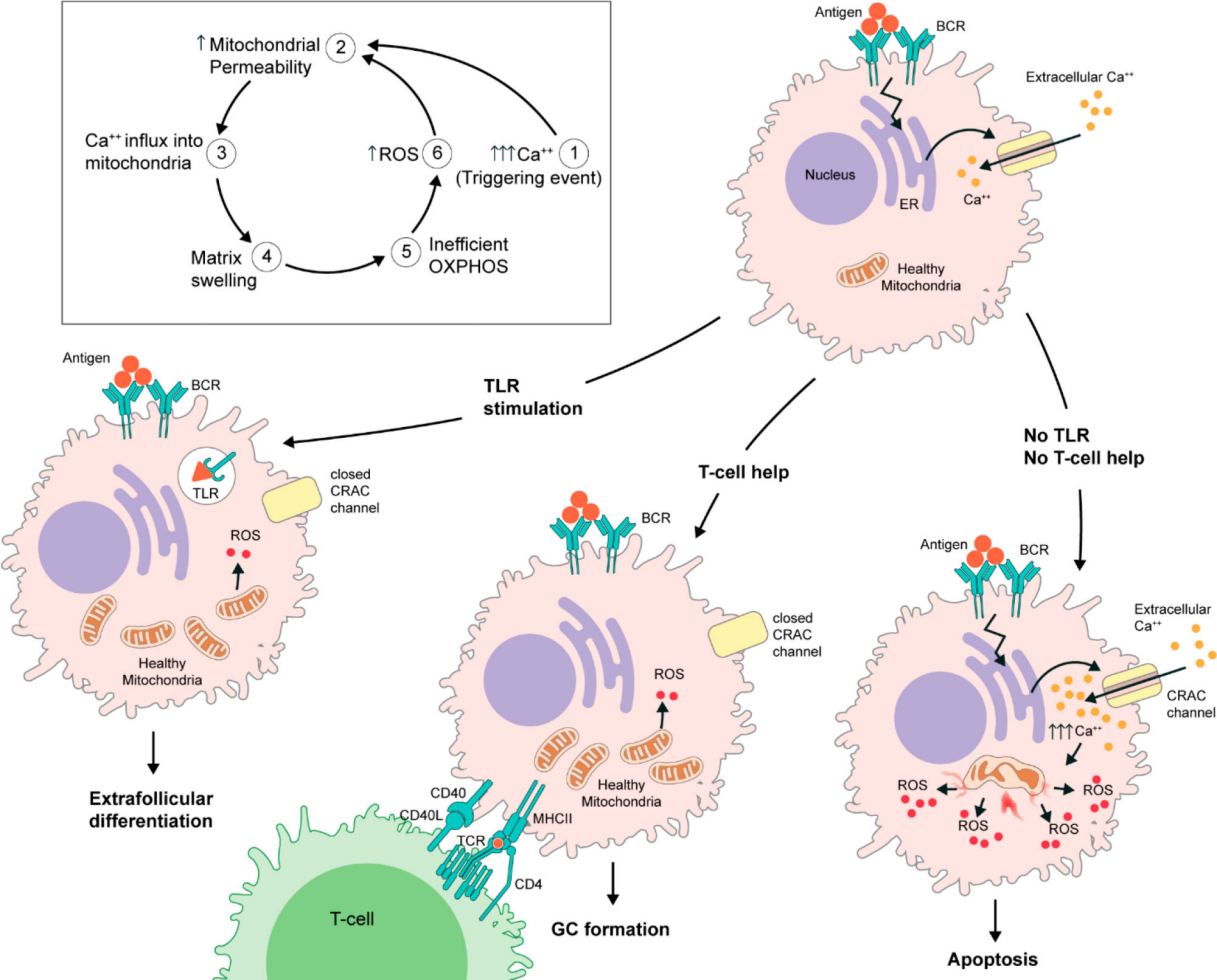
Schematic demonstration of the metabolic clock model of B cell activation and differentiation under the influence of signal one and signal two.
